# Supplementary material for: Delay-dependent contributions of medial temporal lobe regions to episodic memory retrieval
Source: eLife. 2015 Jan 13;4:e05025. doi: 10.7554/eLife.05025 (PMC4337612; doi:10.7554/eLife.05025)
Supplement: Supplementary file 1. — MTL activity estimates for all conditions (related to Figure 2). DOI: http://dx.doi.org/10.7554/eLife.05025.019 [file elife05025s005.docx]

**Supplementary File 1.** MTL activity estimates for all conditions (related to Figure 2).

| **Region** | **Recollection trials** | | **Familiarity trials** | | **Miss trials** | |
| --- | --- | --- | --- | --- | --- | --- |
|  | **Immediate** | **Delayed** | **Immediate** | **Delayed** | **Immediate** | **Delayed** |
| L anterior HF | -0.02 | -0.09 | -0.36 | -0.32 | -0.34 | -0.35 |
| R anterior HF | -0.09 | -0.11 | -0.30 | -0.26 | -0.43 | -0.35 |
| L posterior HF * | 0.23 | 0.06 ^ | -0.07 | 0.07 | -0.06 | -0.09 |
| R posterior HF * | 0.23 | 0.08 ^ | 0.02 | 0.05 | -0.12 | -0.15 |
| L PRC | 0.29 | 0.34 ^ | 0.02 | 0.00 | -0.11 | -0.08 |
| R PRC | 0.08 | 0.04 | -0.15 | -0.10 | -0.20 | -0.13 |
| L PHC | 0.34 | 0.48 | 0.16 | 0.18 | 0.07 | 0.08 |
| R PHC | 0.21 | 0.28 | -0.06 | 0.05 | -0.12 | -0.02 |

Values represent the mean contrast estimate for each trial type (versus implicit baseline). Asterisks (*) denote a significant memory (recollection, familiarity) by delay (immediate, delayed) interaction, *p*<.05. There were no significant memory (familiarity, miss) by delay interactions. Carets (^) denote a significant simple effect of delay, *p*<.05, computed separately for recollection or familiarity.
