# Supplementary material for: Delay-dependent contributions of medial temporal lobe regions to episodic memory retrieval
Source: eLife. 2015 Jan 13;4:e05025. doi: 10.7554/eLife.05025 (PMC4337612; doi:10.7554/eLife.05025)
Supplement: Supplementary file 2. — Table of peak coordinates from the voxel-wise comparison of recollection and familiarity trial activity (related to Figure 3). DOI: http://dx.doi.org/10.7554/eLife.05025.020 [file elife05025s006.docx]

**Supplementary File 2.** Table of peak coordinates from the voxel-wise comparison of recollection and familiarity trial activity (related to Figure 3).

|  |  | **Cluster size** | **Peak statistics** | | **Peak MNI coordinates** | | |
| --- | --- | --- | --- | --- | --- | --- | --- |
| **Region** | **Hem** | **# voxels** | **t** | **z** | **x** | **y** | **z** |
|  |  |  |  |  |  |  |  |
| **Immediate Recollection > Familiarity** | |  |  |  |  |  |  |
| Inferior Parietal Lobule | L | 686 | 9.83 | 5.71 | -36 | -63 | 42 |
| Angular Gyrus | L | -- | 6.88 | 4.76 | -42 | -66 | 36 |
| Supramarginal Gyrus | L | -- | 6.11 | 4.44 | -57 | -57 | 30 |
| Middle Temporal Gyrus | L | 232 | 8.95 | 5.46 | -57 | -45 | -3 |
| -- | L | -- | 5.71 | 4.26 | -63 | -33 | -9 |
| -- | L | -- | 5.43 | 4.12 | -54 | -33 | -9 |
| Precuneus | L | 792 | 8.22 | 5.24 | -12 | -60 | 21 |
| Posterior Cingulate | L | -- | 7.59 | 5.02 | -9 | -57 | 12 |
| Cingulate Gyrus | L | -- | 7.19 | 4.88 | -3 | -42 | 39 |
| Middle Frontal Gyrus | L | 349 | 7.27 | 4.91 | -27 | 18 | 48 |
| -- | L | -- | 6.95 | 4.78 | -21 | 30 | 45 |
| Superior Frontal Gyrus | L | -- | 5.98 | 4.38 | -15 | 39 | 45 |
| Medial Frontal Gyrus | L | 172 | 5.70 | 4.25 | -6 | 48 | -9 |
| -- | L | -- | 4.81 | 3.81 | -3 | 57 | -6 |
| Putamen | R | 45 | 5.65 | 4.23 | 12 | 12 | -6 |
| Inferior Frontal Gyrus | R | -- | 4.19 | 3.46 | 24 | 18 | -18 |
| Putamen | R | -- | 3.76 | 3.19 | 21 | 18 | -9 |
| Posterior Parahippocampal Gyrus | L | 128 | 5.63 | 4.22 | -33 | -36 | -12 |
| -- | L | -- | 5.58 | 4.20 | -36 | -48 | -9 |
| Fusiform Gyrus | L | -- | 5.32 | 4.07 | -42 | -48 | -21 |
| Inferior Frontal Gyrus | L | 85 | 5.48 | 4.15 | -27 | 15 | -21 |
| -- | L | -- | 4.36 | 3.56 | -27 | 24 | -21 |
| Anterior Cingulate | L | -- | 4.22 | 3.47 | -6 | 9 | -12 |
| Precentral Gyrus | R | 43 | 4.69 | 3.74 | 21 | -21 | 66 |
| -- | R | -- | 4.37 | 3.56 | 21 | -27 | 60 |
| Superior Temporal Gyrus | R | 44 | 4.68 | 3.73 | 57 | -33 | 9 |
| -- | R | -- | 4.55 | 3.67 | 51 | -27 | 3 |
| Superior Frontal Gyrus | L | 54 | 4.60 | 3.69 | -6 | 60 | 27 |
| Medial Frontal Gyrus | L | -- | 4.43 | 3.60 | -3 | 66 | 21 |
| Superior Frontal Gyrus | L | -- | 4.36 | 3.56 | -15 | 60 | 21 |
|  |  |  |  |  |  |  |  |
| **Delayed Recollection > Familiarity** |  |  |  |  |  |  |  |
| Precuneus | L | 370 | 6.53 | 4.62 | -42 | -78 | 42 |
| Angular Gyrus | L | -- | 5.16 | 3.99 | -48 | -72 | 30 |
| Superior Parietal Lobule | L | -- | 4.57 | 3.68 | -36 | -69 | 54 |
| Middle Frontal Gyrus | L | 82 | 5.96 | 4.37 | -21 | 27 | 45 |
| Inferior Frontal Gyrus | L | 123 | 5.84 | 4.32 | -51 | 30 | 3 |
| -- | L | -- | 5.00 | 3.91 | -45 | 27 | -12 |
| -- | L | -- | 4.77 | 3.79 | -36 | 30 | -15 |
| Posterior Cingulate | L | 209 | 5.69 | 4.25 | -6 | -45 | 36 |
| -- | L | -- | 5.35 | 4.09 | -9 | -54 | 15 |
| Precuneus | L | -- | 5.13 | 3.98 | -6 | -63 | 30 |
| Posterior Parahippocampal Gyrus | L | 38 | 5.31 | 4.07 | -27 | -30 | -18 |
| Superior Frontal Gyrus | L | 93 | 5.18 | 4.00 | -12 | 63 | 24 |
| Medial Frontal Gyrus | L | -- | 4.58 | 3.68 | -9 | 69 | 9 |
| Superior Frontal Gyrus | L | -- | 4.44 | 3.60 | -9 | 66 | 33 |
| Middle Frontal Gyrus | L | 51 | 4.96 | 3.89 | -39 | 12 | 48 |
| -- | L | -- | 4.92 | 3.86 | -39 | 9 | 60 |
| -- | L | -- | 4.28 | 3.51 | -45 | 24 | 51 |
| Middle Temporal Gyrus | L | 55 | 4.54 | 3.66 | -57 | -33 | -9 |
| -- | L | -- | 4.24 | 3.48 | -63 | -21 | -18 |
| Inferior Temporal Gyrus | L | -- | 3.91 | 3.28 | -54 | -18 | -21 |

Note: Peaks are reported for clusters surviving a voxel-wise threshold of *p*<.001 and cluster-corrected *p*<.05 (at least 36 voxels). Regions are labeled with the nearest label from the Talairach Daemon. For each cluster, up to 3 subpeaks at least 12 mm apart are reported. Dashes in the # voxels column indicate that the subpeak is from the same cluster as the previous peak. Dashes in the Region column indicate that this subpeak is assigned the same label as the previous peak from that cluster. Hem=hemisphere, L=left, R=right.
